# The Mobile Insulin Titration Intervention (MITI) for Insulin Glargine Titration in an Urban, Low-Income Population: Randomized Controlled Trial Protocol

**DOI:** 10.2196/resprot.4206

**Published:** 2015-03-13

**Authors:** Natalie Levy, Victoria Moynihan, Annielyn Nilo, Karyn Singer, Lidia S Bernik, Mary-Ann Etiebet, Yixin Fang, James Cho, Sundar Natarajan

**Affiliations:** ^1^Division of General Internal Medicine and Clinical InnovationDepartment of MedicineNew York University School of MedicineNew York, NYUnited States; ^2^Bellevue Hospital CenterNew York, NYUnited States; ^3^Urban Health PlanNew York, NYUnited States; ^4^Mount Sinai Health SystemNew York, NYUnited States; ^5^New York City Health and Hospitals CorporationNew York, NYUnited States; ^6^Division of BiostatisticsDepartment of Population HealthNew York University School of MedicineNew York, NYUnited States; ^7^Department of Population HealthNew York University School of MedicineNew York, NYUnited States; ^8^Department of Veterans AffairsNew York Harbor Healthcare SystemNew York, NYUnited States

**Keywords:** patient care management, delivery of care, health care disparities, telemedicine, remote consultation

## Abstract

**Background:**

Patients on insulin glargine typically visit a clinician to obtain advice on how to adjust their insulin dose. These multiple clinic visits can be costly and time-consuming, particularly for low-income patients. It may be feasible to achieve insulin titration through text messages and phone calls with patients instead of face-to-face clinic visits.

**Objective:**

The objectives of this study are to (1) evaluate if the Mobile Insulin Titration Intervention (MITI) is clinically effective by helping patients reach their optimal dose of insulin glargine, (2) determine if the intervention is feasible within the setting and population, (3) assess patient satisfaction with the intervention, and (4) measure the costs associated with this intervention.

**Methods:**

This is a pilot study evaluating an approach to insulin titration using text messages and phone calls among patients with insulin-dependent type 2 diabetes in the outpatient medical clinic of Bellevue Hospital Center, a safety-net hospital in New York City. Patients will be randomized in a 1:1 ratio to either the MITI arm (texting/phone call intervention) or the usual-care arm (in-person clinic visits). Using a Web-based platform, weekday text messages will be sent to patients in the MITI arm, asking them to text back their fasting blood glucose values. In addition to daily reviews for alarm values, a clinician will rereview the texted values weekly, consult our physician-approved titration algorithm, and call the patients with advice on how to adjust their insulin dose. The primary outcome will be whether or not a patient reaches his/her optimal dose of insulin glargine within 12 weeks.

**Results:**

Recruitment for this study occurred between June 2013 and December 2014. We are continuing to collect intervention and follow-up data from our patients who are currently enrolled. The results of our data analysis are expected to be available in 2015.

**Conclusions:**

This study explores the use of widely-available text messaging and voice technologies for insulin titration. We aim to show that remote insulin titration is clinically effective, feasible, satisfactory, and cost saving for low-income patients in a busy, urban clinic.

**Trial Registration:**

Trial Registration: Clinicaltrials.gov NCT01879579; http://clinicaltrials.gov/ct2/show/NCT01879579 (Archived by WebCite at http://www.webcitation.org/6WUEgjZUO).

## Introduction

### Background

Diabetes disproportionately affects the poor and uninsured, who are more likely to suffer the severe health consequences of uncontrolled diabetes, including heart disease, death, stroke, blindness, renal failure, and nontraumatic lower limb amputations [1].

Diabetes care is quite complex. Patients often need to learn self-management (eg, monitoring home blood glucose), make multiple lifestyle changes (eg, diet, exercise), follow complex medication regimens, and attend multiple clinic appointments (eg, primary care providers, diabetic educators, specialists). In public hospitals and clinics that serve low-income populations, these appointment slots can be few and often occur during patients’ work hours. Patients have to miss work, make arrangements for the children in their care, and arrange transportation to the clinic. These logistical and cost-related barriers to accessing clinic care contribute to the socioeconomic disparity in diabetes management [2,3,4,5].

Bellevue Hospital Center, located in New York City (NYC), primarily treats low-income, ethnically diverse patients [6]. The prevalence rate of diabetes in the hospital’s Adult Primary Care Center (APCC) is 15%, compared to the rate of 10.5% in NYC and 9% in the US (hospital prevalence rate obtained from internal hospital database, HHC Patient Registry for Proactive Care) [6]. Many of these patients are advised to start insulin therapy, in accordance with the standard for diabetes care. Proper insulin treatment involves multiple steps, including the need for patients to communicate blood glucose values to a clinician who then adjusts the patient’s insulin dose accordingly. Traditionally, this exchange of blood glucose data is achieved through face-to-face appointments with a clinician [7-11]. Given the many challenges low-income patients face attending frequent appointments, patients may struggle to have their insulin titrated regularly and the process of optimizing their dose is prolonged [9,12].

Mobile technology, especially mobile phones, may help alleviate the logistical barriers to care. Currently, 90% of US adults own a mobile phone, and 58% own mobile phones with advanced operating systems (smartphones). While only 47% of low-income adults own an advanced mobile phone, 84% of low-income adults own any mobile phone (basic or advanced) [13]. Text messaging and voice calls are available on most basic mobile phones, making interventions using basic mobile phone technology a viable option for low-income populations.

Several studies have used text messaging to help patients manage their diabetes care, even among low-income populations [8,14-18]. Text messages have been used successfully to remind patients to carry out self-care (eg, monitoring home blood glucose levels) and to transmit this data to the clinician, thus ensuring that a home blood glucose log is available at the time of the next in-person visit. We also found studies where clinicians adjusted insulin doses remotely. In these studies, patients sent their blood glucose values to their clinicians by accessing the Internet. Clinicians could respond by uploading their advice via the Internet or through text message. These studies demonstrate the feasibility of the remote exchange of both blood glucose values and insulin dosage advice. However, they required patients to have access to the Internet and, in some cases, to navigate a website [8,17,19].

The Mobile Insulin Titration Intervention (MITI) incorporates the strengths of the studies above by using text messages as a prompt to remind patients to check their home blood glucose values and by allowing the remote exchange of actionable data, namely the receipt of the values and the transmission of titration advice. MITI builds upon the above studies by tailoring these interventions to low-income patients, requiring only texting capabilities to send the blood glucose values and a simple phone call to receive titration instructions. This intervention requires only a low-cost, basic-feature mobile phone, not an advanced mobile phone or Internet access, which our patients often do not have.

We designed the intervention to include both text messages and phone calls for insulin titration, rather than one or the other. Text messages act as a reminder to patients to check their blood glucose levels and can be automated to be sent to the patient at a given time of day. Text messaging also provides a simple and quick way for patients to send their blood glucose data to the clinician from any location, given their phones are able to send text messages. We included phone calls for the adjustment of the insulin dose as a balanced approach between automated instructions (ie, insulin dosage sent via text, email, or Internet portal) and the personal nature of the in-person clinic appointment. Weekly phone calls give the patient an opportunity to discuss their treatment progress in real time with a clinician, while allowing the clinician to leave a voicemail with instructions if the patient is not available. This intervention is designed to alleviate the burden of in-person titration visits in a pragmatic manner, using basic technology available to our patient population.

### Objectives

The objectives of this study are to (1) evaluate if MITI is clinically effective by helping patients reach their optimal dose of insulin glargine (defined in the *Outcome Measures* section), (2) determine if the intervention is feasible within the setting and population, (3) assess patient satisfaction with the intervention, and (4) measure the costs associated with this intervention.

## Methods

### Overview

The MITI study is a randomized controlled trial to evaluate a mobile phone intervention for insulin glargine titration. This trial has been registered at ClinicalTrials.gov (NCT01879579). We will recruit patients from Bellevue Hospital Center’s Adult Primary Care Center who have type 2 diabetes and require insulin glargine titration. We will focus on glargine because it is the type of insulin used in our formulary. Using a parallel study design, patients will be randomized into either the MITI (intervention) arm or the usual-care (control) arm. Patients in the MITI arm will send their fasting blood glucose levels each weekday via text messages and have their insulin dose adjusted during phone calls with a diabetes nurse educator using a physician-approved algorithm. Patients in the usual-care arm will continue to receive their usual care, which includes scheduled clinic appointments to review daily blood glucose levels and to adjust their insulin dose. The primary outcome will be whether or not a patient reaches his/her optimal insulin glargine dose within 12 weeks.

### Setting

Bellevue Hospital Center is a large tertiary care hospital within New York City’s Health and Hospitals Corporation, the largest public hospital system in the United States and one of the largest safety-net providers. Bellevue Hospital’s APCC cares for an ethnically diverse patient population, where 71% of the patients are nonwhite (41% Hispanic, 24% black, and 6% Asian). In the APCC, 76% of outpatient clinic visits are for patients who are either uninsured (31%) or have Medicaid (45%) [6]. Patients in this study will be recruited from the APCC, where the prevalence rate of diabetes is 15%.

### Participants

We will recruit patients with diabetes who present to the Adult Primary Care Center. The inclusion criteria are as follows: (1) patients who are initiating insulin glargine treatment or require the titration of their existing insulin glargine dose, (2) English or Spanish speaking, (3) most recent hemoglobin A1c (HbA1c) value is ≥8%, (4) able and willing to inject insulin, and (5) able and willing to provide informed consent. We will exclude patients who meet the following criteria: (1) on short-acting insulin, (2) on systemic glucocorticoids, (3) with sustained serum creatinine of ≥1.5 mg/dL for men and ≥1.4 mg/dL for women, (4) with documented hypoglycemia unawareness, and (5) diagnosed with type 1 diabetes.

### Outcome Measures

The primary outcome will be whether or not a patient reaches his/her optimal insulin glargine dose within 12 weeks of enrolling in the study. Optimal insulin glargine dose is defined as the dose at which the patient has at least one fasting blood glucose value within the range of 80 to 130 mg/dL inclusive, or the maximum dose that can be safely administered to the patient. The research staff will record whether a patient has reached their optimal insulin dose at the time of the patient’s weekly titration phone call (if in the MITI arm) or clinic appointment (if in the usual-care arm).

We hypothesize that the MITI arm will have a greater proportion of patients who reach their optimal insulin dose than the usual-care arm. Other clinical effectiveness outcomes include the time taken to reach optimal dose, the incidence of hypoglycemia, and the change in HbA1c levels between baseline and 3 months.

Feasibility measures include patients’ text message response rate, ability of the diabetes nurse educator to reach patients for insulin titration, and the time spent by the diabetes nurse educator on the intervention.

We will measure patient treatment satisfaction at baseline and 3 months after study enrollment using the Diabetes Treatment Satisfaction Questionnaire. We will use an additional questionnaire—the “change” version of the Diabetes Treatment Satisfaction Questionnaire—to measure the change in the patient’s treatment satisfaction between baseline and 3 months [20]. We will also use a semistructured interview to gather qualitative feedback from patients in the MITI arm. This will be administered when the patient has completed the intervention—by reaching their optimal insulin dose or when 12 weeks elapse.  

We will collect data on the costs of insulin titration to compare the intervention to the established standard of care in the clinic. These outcomes include the time spent by patients traveling to the clinic, time spent in the waiting room prior to appointments, number and duration of insulin titration appointments, patient co-pays, and patient health care utilization (ie, the number of noninsulin-related medical clinic visits made at Bellevue during the 12-week study period).

### Interventions

#### Usual-Care

**Figure 1:**
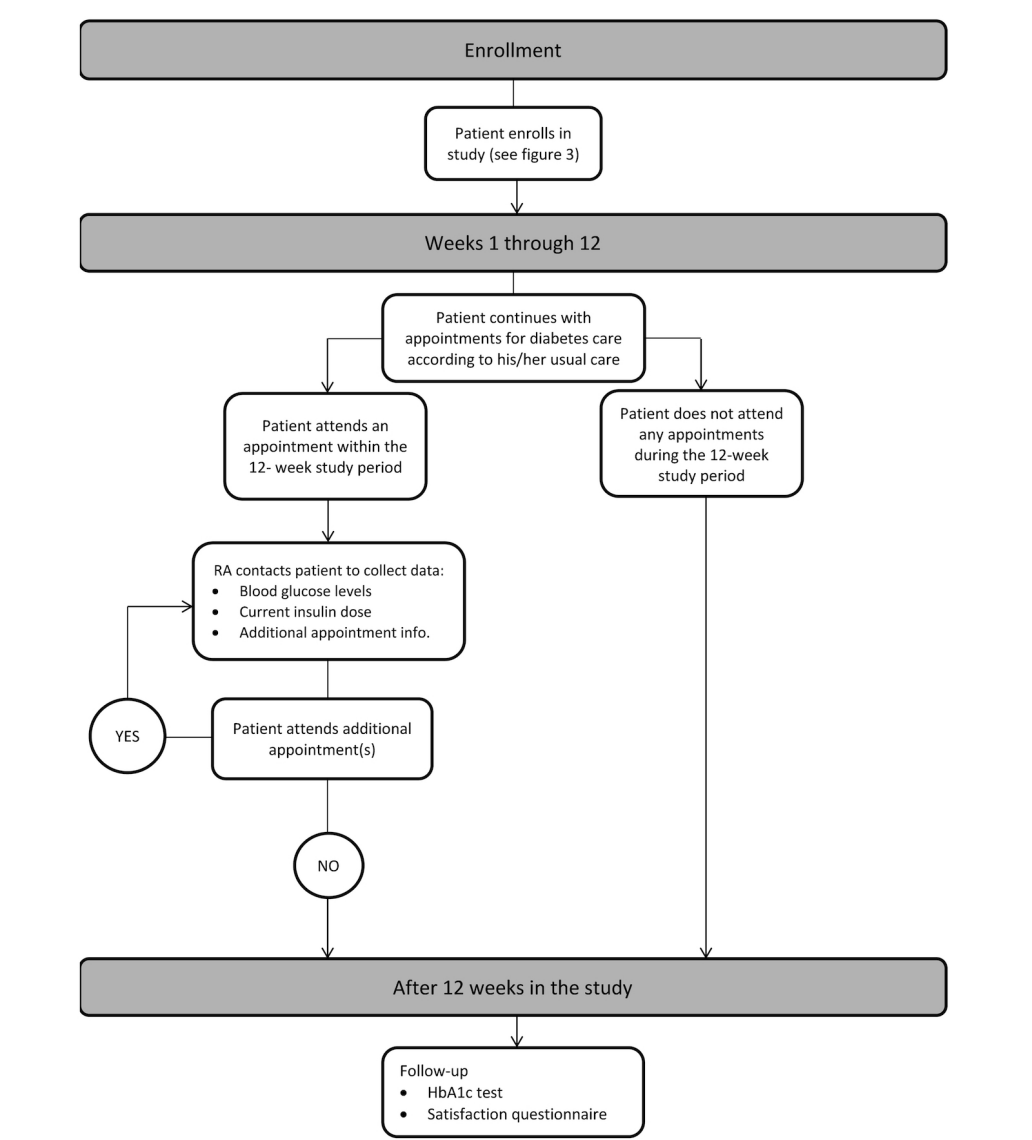
Usual-care flowchart.

Patients in the usual-care arm will continue with the treatment plan and appointments decided upon with their clinician prior to enrolling in the study (see Figure 1). According to the clinic’s current practice, a clinician reviews the patient’s fasting blood glucose log during appointments and titrates the insulin dose. Our research assistant (RA) will collect data, such as fasting blood glucose readings, insulin dosage adjustments, and appointment duration, from patients after appointments, in-person or by phone, as well as appointment duration from clinicians. These patients will continue to visit their primary care providers and have routine HbA1c tests according to the standard of care.

#### Mobile Insulin Titration Intervention

Patients in the MITI arm will sign up for a Web-based health management platform in the clinic with the assistance of the research staff. The platform allows patients and clinicians to communicate via text messaging. Patients will receive a text message each weekday from the platform requesting the patient’ s fasting blood glucose level for that day. The messages will be automatically delivered each morning at a time prespecified by the patient, in either English or Spanish. Patients will respond via text message with their fasting blood glucose levels. Mobile phones can be provided to any patient who is otherwise eligible but does not have a mobile phone. It is implicit that a patient must be able to operate a mobile phone to participate in the study.

The diabetes nurse educator will check patients’ responses on the secure Web portal each weekday and call any patient reporting alarm values (ie, a blood glucose level <80 mg/dL or >400 mg/dL). Each Thursday afternoon, the diabetes nurse educator will call the patients to adjust their insulin dose according to a titration algorithm developed by physicians and nurses on the study team. The diabetes nurse educator may leave a voicemail with insulin titration instructions. If the patient cannot be contacted by phone, the diabetes nurse educator may call the patient’s emergency contact upon her discretion. Another clinician may check text responses and make titration phone calls if the diabetes nurse educator is not available. If Thursday is a holiday, the titration calls can take place on another weekday.

**Figure 2:**
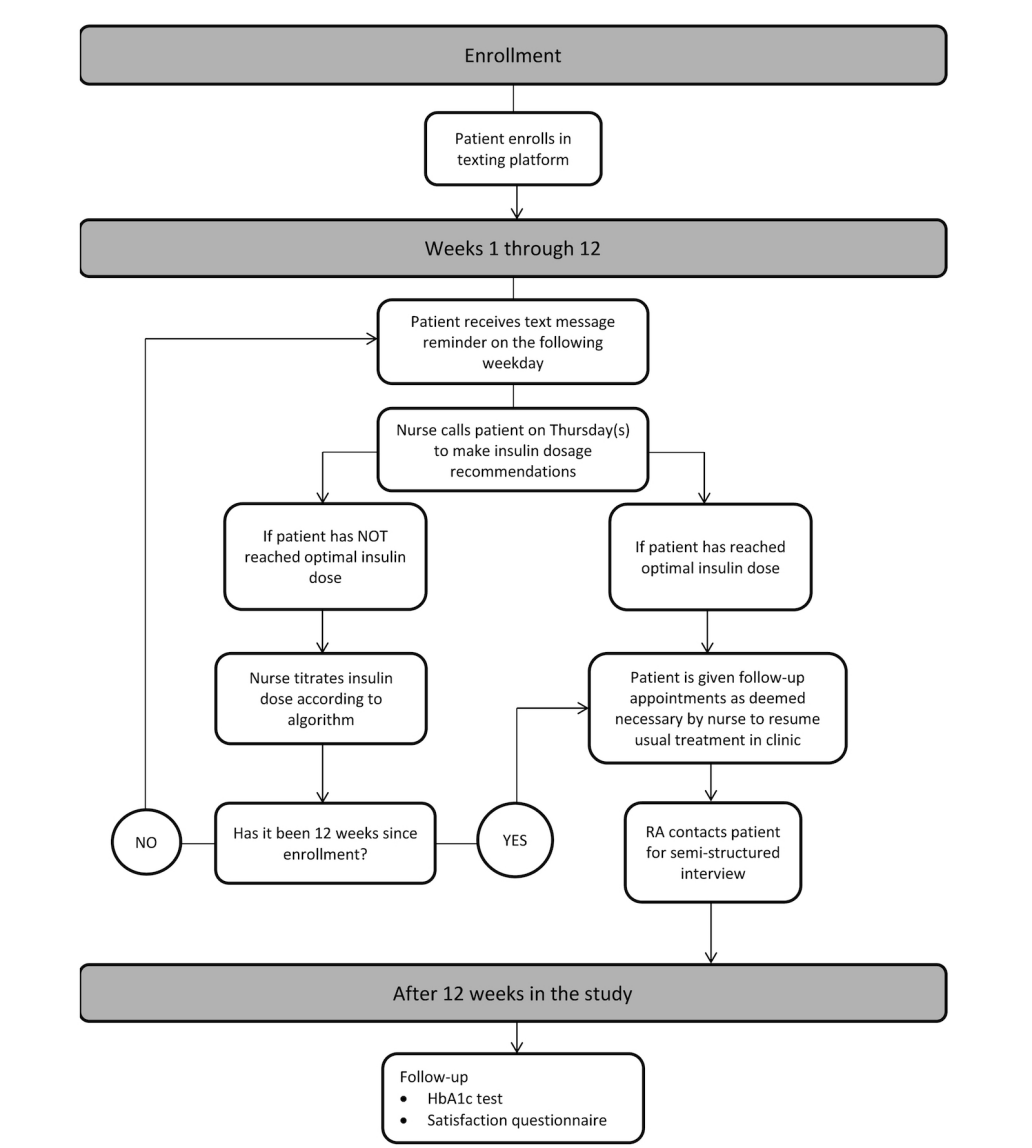
Intervention (MITI) flowchart.

This protocol of weekday text messages and weekly phone calls will continue for up to 12 weeks (see Figure 2), until the patient reaches their optimal insulin dose, or the patient chooses to withdraw from the study. Patients may attend appointments with their primary care provider during the intervention, but will not need to attend appointments specifically for diabetes management (eg, high HbA1c clinic or diabetes nurse educator appointments) within the primary care clinic. After completing the protocol, the patient will be transferred back to usual care in the clinic. The research team will arrange for follow-up appointments with the patient’s primary care provider and, if appropriate, appointments with the diabetes nurse educator or high HbA1c clinic, so that the patient can resume their diabetes care through standard clinic visits.

#### Follow-Up

Patients in both arms will be contacted by the RA at approximately 12 weeks in the study to remind them of their routine HbA1c test. The RA will also arrange to administer the Diabetes Treatment Satisfaction Questionnaire, either when the patient is present in the clinic for their blood test or via phone.

### Implementation Challenges

During this pilot study, we refined our technological approach because our initial health management platform could not send text messages to prepaid mobile phones. Patients who had prepaid mobile phones either continued to attend in-person clinic appointments for insulin titration or were provided a mobile phone to use for the duration of the study. We resolved this issue in 2014 when we switched to a different health management platform that could send text messages to any mobile phone.

Prior to May 2014, patients were stratified by whether the patient was initiating insulin therapy or having their existing insulin dose titrated, and by HbA1c level (8-11% or >11%). We removed the HbA1c stratification after finding that not all patients had an HbA1c value available in their medical record at the time of enrollment.

### Intervention Standardization and Fidelity

To ensure that all patients receive the same level of care during the intervention, the diabetes nurse educator will use a script outlining what must be covered during an insulin titration phone call. The nurse will also use scripts to leave titration instructions via voicemail and for speaking with, and leaving voicemails for, the patient’s emergency contact. These scripts were implemented in May 2014. The nurse can consult the principal investigator in real time if any event arises with a patient that is not outlined in the study protocol.

### Sample Size (Power Analysis)

Our sample size is based on the hypothesis that the proportion of patients in the MITI group who reach their optimal insulin dose within 12 weeks will be significantly greater that the proportion of patients in the usual-care group who reach this goal. Based on the experience of clinicians treating our patient population at our study site, we expect that, at most, 50% of patients in the usual-care arm will reach their optimal insulin dose by 12 weeks. We expect at least 80% of patients in the MITI arm to reach this goal, since this arm will have weekly insulin titration phone calls, and frequent titration is associated with improved glycemic control [10,21,22]. Using Fisher's exact test with a 2-sided type I error rate of 5% to achieve at least 80% power, we need 44 subjects per group to detect a difference of 30% between 80% and 50%. Assuming a 10% drop-out rate by week 12, we need a total of 98 patients (49 per group).

### Recruitment, Randomization, and Retention

**Figure 3:**
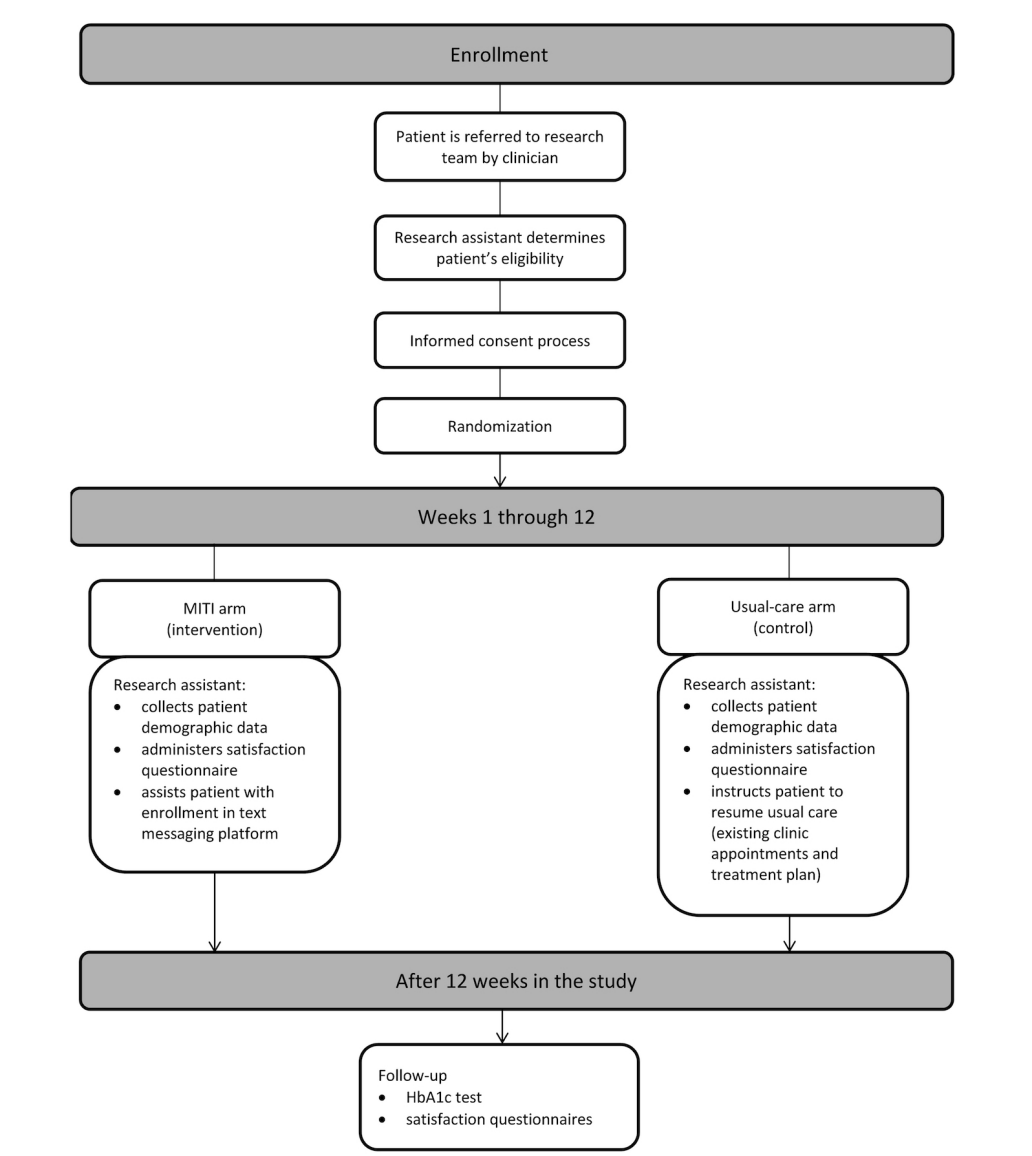
Enrollment flowchart.

We will recruit patients from the Adult Primary Care Center. Clinicians can alert the research study staff of any patient who is interested in the study and may be eligible (see Figure 3). The RA will also review the clinic’s electronic medical records to identify patients that may be eligible for the study. The RA will screen the patient for eligibility, explain the study, and provide them with a consent form to read and sign in person at the Adult Primary Care Center. All patients must provide informed consent before participating in the study protocol.

Since patients will be randomized sequentially at the time they provide informed consent, we will use block randomization (block size of 4) to make sure the number of patients in each arm is approximately balanced at any time point during the study. Patients will be stratified by whether the patient is initiating insulin therapy or having their existing insulin dose titrated. The random allocation sequence will be computer-generated by a coinvestigator and concealed in presealed envelopes. The patient’s arm assignment will be revealed to the patient and research team at the time of study enrollment by the RA. The research team and clinicians will not be blinded since the intervention requires that they alter the patient’s treatment plan or, in the case of the usual-care arm, collect data from clinicians and patients regarding their appointments.

At the time of enrollment, patients will receive a US $10 MetroCard to be used for travel to and from the clinic. Patients will receive a glucometer and a supply of test strips to ensure that they can test their blood glucose levels for the duration of the study. At approximately 12 weeks into the study, the research assistant will contact patients by phone to remind them to return to the clinic for their 3-month HbA1c blood test and complete the satisfaction questionnaire. Patients will receive an additional US $10 MetroCard.

### Data Monitoring

A data and safety monitoring board is designated to meet quarterly, or sooner if necessary, to discuss any potential safety issues. In particular, the study team and the board will review any cases of hypoglycemia or hyperglycemia that occur among our patients (to assess the safety of our titration algorithm) and our ability to reach patients by phone (to ensure that our patients are being monitored regularly during the intervention).

### Data Analysis

First, we will summarize all the baseline and follow-up measures using means and standard deviation (SD), medians and interquartile range (IQR), or frequencies, and then compare them between the two arms. Second, we will test if baseline characteristics (eg, demographic measures and baseline HbA1c levels) are balanced between the two randomized arms. Third, to evaluate the intervention effects, we will use the chi-square test or Fisher’s exact test for categorical outcomes (eg, whether or not a patient reaches optimal dose), Student’s *t* test or Wilcoxon rank-sum test for continuous outcomes (eg, change in HbA1c levels, rate of hypoglycemia, scores on the Diabetes Treatment Satisfaction Questionnaire, number and duration of titration appointments, and patient health care utilization), and log-rank test for time to reach optimal dose. Fourth, we will conduct multiple linear regression analyses for continuous outcomes and multiple logistic regression analyses for categorical outcomes to further evaluate the intervention effects, adjusting for some baseline characteristics and/or their interactions with the treatment assignment. At this stage, we will conduct multiple imputation to deal with the missing data problem. Finally, we will conduct descriptive analyses for other secondary outcomes, such as feasibility outcomes, patient travel time and waiting room time, and patient co-pays. We will also review the content of semistructured interviews to identify common themes in patient feedback.

## Results

Recruitment for this study occurred between June 2013 and December 2014. We are continuing to collect intervention and follow-up data from our patients who are currently enrolled. The results of our data analysis are expected to be available in 2015.

## Discussion

The MITI study builds upon lessons learned in previous interventions and presents an innovative approach to insulin titration. It addresses the need for further research on mobile health interventions for diabetes and other chronic diseases. Research is especially needed in low-income populations who face numerous challenges in managing chronic illness.

The MITI study has a few limitations. We anticipate a small sample size for this study, which will limit our statistical power. We also have to consider volunteer bias, since those patients who choose to participate may not be representative of the population of patients we are seeking to treat (ie, those with type 2 diabetes in need of insulin titration).

While we use a strong study design (randomized controlled trial), our study is not blinded. Since our intervention directly affects the treatment our patients receive in the clinic, the patient’s primary care provider and their other clinicians may become aware that their patient is participating in the study. In addition, those who are allocated to the usual-care arm will be contacted periodically by the research staff for data collection. While this data collection is necessary to measure certain outcomes for both study arms, we cannot rule out that patients may alter their behavior, since they are aware that their treatment progress is being monitored (ie, observer bias).

## Multimedia Appendix 1

CONSORT-EHEALTH Checklist V1.6.2 [23].

## Abbreviations

APCC: Adult Primary Care Center
HbA1c: hemoglobin A1c
IQR: interquartile range
MITI: Mobile Insulin Titration Intervention
NCATS: National Center for the Advancement of Translational Science
NYC: New York City
NYU-HHC CTSI: New York University-Health and Hospitals Corporation Clinical and Translational Science Institute
RA: research assistant
