# Addendum to: The Mobile Insulin Titration Intervention (MITI) for Insulin Glargine Titration in an Urban, Low-Income Population: Randomized Controlled Trial Protocol

**DOI:** 10.2196/resprot.5403

**Published:** 2015-12-21

**Authors:** Natalie Levy, Victoria Moynihan, Annielyn Nilo, Karyn Singer, Lidia S Bernik, Mary-Ann Etiebet, Yixin Fang, James Cho, Sundar Natarajan

**Affiliations:** ^1^ Division of General Internal Medicine and Clinical Innovation Department of Medicine New York University School of Medicine New York, NY United States; ^2^ Bellevue Hospital Center New York, NY United States; ^3^ Gouverneur Diagnostic and Treatment Center New York, NY United States; ^4^ Department of Healthcare Policy and Research Weill Cornell Medical College New York, NY United States; ^5^ Division of Biostatistics Department of Population Health New York University School of Medicine New York, NY United States; ^6^ Department of Population Health New York University School of Medicine New York, NY United States; ^7^ Department of Veterans Affairs New York Harbor Healthcare System New York, NY United States

The authors of “The Mobile Insulin Titration Intervention (MITI) for Insulin Glargine Titration in an Urban, Low-Income Population: Randomized Controlled Trial Protocol” (JMIR Res Protoc 2015;4[1]:e31) are adding their insulin glargine titration algorithm to this protocol paper. The authors feel that readers may be interested in seeing the algorithm used in the trial. The algorithm was added as Multimedia Appendix 2 to the paper and the article was resubmitted to Pubmed Central.

 

## Multimedia Appendix 2

Insulin titration algorithm.

