# Supplementary material for: The Mobile Insulin Titration Intervention (MITI) for Insulin Glargine Titration in an Urban, Low-Income Population: Randomized Controlled Trial Protocol
Source: JMIR Res Protoc. 2015 Mar 13;4(1):e31. doi: 10.2196/resprot.4206 (PMC4381814; doi:10.2196/resprot.4206)
Supplement: Supplementary file 1 [file resprot_v4i1e31_app1.pdf]

# CONSORT-EHEALTH Checklist V1.6.2 Report

**(based on CONSORT-EHEALTH V1.6), available at [http://tinyurl.com/consort-ehealth-v1-6].**

4206

**Date completed**

1/5/2015 17:17:32

**by**

Victoria Moynihan

The Mobile Insulin Titration Intervention (MITI) Study: A randomized controlled trial using mobile phones for insulin glargine titration in an urban, low-income population.

## TITLE

**1a-i) Identify the mode of delivery in the title**

Yes, the study uses mobile phones.
"The Mobile Insulin Titration Intervention (MITI) Study: A randomized controlled trial using mobile phones for insulin glargine titration in an urban, low-income population."

**1a-ii) Non-web-based components or important co-interventions in title**

**1a-iii) Primary condition or target group in the title**

Yes, the target group is patients who require insulin glargine titration."The Mobile Insulin Titration Intervention (MITI) Study: A randomized controlled trial using mobile phones for insulin glargine titration in an urban, low-income population."

## ABSTRACT

**1b-i) Key features/functionalities/components of the intervention and comparator in the METHODS section of the ABSTRACT**

Yes, the abstract summarizes the trial design and discussion. This is a study protocol and the results of the study are not yet available.

"Introduction
Patients on insulin glargine typically visit a clinician to obtain advice on how to adjust their insulin dose. These multiple clinic visits can be costly and time-consuming, particularly for low-income patients. It may be feasible to achieve insulin titration through text messages and phone calls with patients instead of face-to-face clinic visits.

Methods
This is a pilot study evaluating an approach to insulin titration using text messages and phone calls among patients with insulin-dependent type 2 diabetes in the outpatient medical clinic of Bellevue Hospital Center, a safety-net hospital in New York City. Patients are randomized in a 1:1 ratio to either the MITI arm (texting/phone call intervention) or the usual care arm (in-person clinic visits). Using a web-based platform, weekday text messages are sent to patients in the MITI arm, asking them to text back their morning fasting blood glucose values. In addition to daily reviews for alarm values, a clinician re-reviews the texted values weekly, consults our physician-approved titration algorithm, and calls the patients with advice on how to adjust the insulin dose. The primary outcome is whether or not a patient reaches his/her optimal dose of insulin glargine within 12 weeks.

Discussion
This study explores the use of widely-available text messaging and voice technologies for insulin titration. We aim to show that remote insulin titration is clinically effective, feasible, satisfactory and cost saving for low-income patients in a busy, urban clinic."

**1b-ii) Level of human involvement in the METHODS section of the ABSTRACT**

**1b-iii) Open vs. closed, web-based (self-assessment) vs. face-to-face assessments in the METHODS section of the ABSTRACT**

**1b-iv) RESULTS section in abstract must contain use data**

**1b-v) CONCLUSIONS/DISCUSSION in abstract for negative trials**

## INTRODUCTION

**2a-i) Problem and the type of system/solution**

Yes

"Introduction

Background

Diabetes disproportionately affects the poor and uninsured, who are more likely to suffer the severe health consequences of uncontrolled diabetes (including heart disease, death, stroke, blindness, renal failure, and non-traumatic lower limb amputations) [1].
Diabetes care is quite complex. Patients often need to learn self-management (including monitoring home blood glucose), make multiple lifestyle changes (diet, exercise), follow complex medication regimens, and attend multiple clinic visits (primary care providers, diabetic educators, specialists). In public hospitals and clinics that serve low-income populations, these appointment slots can be few and often occur during patients' work hours. Patients have to miss work, make arrangements for the children in their care, and arrange transportation to the clinic. These logistical and cost-related barriers to accessing clinic care contribute to the socioeconomic disparity in diabetes management [2, 3, 4, 5].
Bellevue Hospital Center, located in New York City, primarily treats low-income, uninsured patients [6]. The prevalence rate of diabetes in the hospital's Adult Primary Care Center (APCC) is 15%, compared to the rate of 10.5% in NYC and 9% in the U.S. (hospital prevalence rate obtained from internal hospital database, HHC Patient Registry for Proactive Care) [6]. Many of these patients are advised to start insulin therapy, in accordance with the standard for diabetes care. Proper insulin treatment involves multiple steps, including the need for patients to communicate blood glucose value to a clinician who then adjusts the patient's insulin dose accordingly. This exchange of blood glucose data is typically achieved through face-to-face appointments with a clinician. Given the many challenges low-income patients face attending frequent appointments, patients may struggle to have their insulin titrated regularly and the process of optimizing their dose is prolonged.
Mobile technology, especially mobile phones may help alleviate the logistical barriers to care. Currently, 90% of U.S. adults own a mobile phone, 58% own smartphones. While only 47% of low-income adults own a smart phone, 84% of low income adults own any mobile phone [7]. Text messaging and voice calls are available on most basic phones making interventions using basic mobile phone technology a viable option for low-income populations. Several studies have used text messaging to help patients manage their diabetes care, even among low-income populations [8-13]. Text messages have been used successfully to remind patients to carry out self-care (for example, monitoring home blood glucose levels) and to transmit this data to the clinician (thus ensuring that a home blood glucose log is available at the time of the next in-person visit). We also found studies where clinicians adjusted insulin doses remotely. In these studies, patients sent their blood glucose values to their clinician by accessing a website. Clinicians could respond by uploading their advice to the website or through text message. These studies demonstrate the feasibility of the remote exchange of both blood glucose values and insulin dosage advice. However, they required patients to have access to the internet and navigate a website [9, 12, 14].
MITI incorporates the strengths of the studies above by using text messages as a prompt to remind patients to check their home blood glucose values and by allowing the remote exchange of actionable data (namely the receipt of the values and the transmission of titration advice). MITI builds upon the above studies by tailoring these interventions to low-income patients requiring only texting capabilities to send the blood glucose values, and a simple phone call to receive titration instructions. This intervention requires only a low-cost basic mobile phone, not a smart phone or internet access, which our patients often do not have."

**2a-ii) Scientific background, rationale: What is known about the (type of) system**

Yes

"Mobile technology, especially mobile phones may help alleviate the logistical barriers to care. Currently, 90% of U.S. adults own a mobile phone, 58% own smartphones. While only 47% of low-income adults own a smart phone, 84% of low income adults own any mobile phone [7]. Text messaging and voice calls are available on most basic phones making interventions using basic mobile phone technology a viable option for low-income populations. Several studies have used text messaging to help patients manage their diabetes care, even among low-income populations [8-13]. Text messages have been used successfully to remind patients to carry out self-care (for example, monitoring home blood glucose levels) and to transmit this data to the clinician (thus ensuring that a home blood glucose log is available at the time of the next in-person visit). We also found studies where clinicians adjusted insulin doses remotely. In these studies, patients sent their blood glucose values to their clinician by accessing a website. Clinicians could respond by uploading their advice to the website or through text message. These studies demonstrate the feasibility of the remote exchange of both blood glucose values and insulin dosage advice. However, they required patients to have access to the internet and navigate a website [9, 12, 14].
MITI incorporates the strengths of the studies above by using text messages as a prompt to remind patients to check their home blood glucose values and by allowing the remote exchange of actionable data (namely the receipt of the values and the transmission of titration advice). MITI builds upon the above studies by tailoring these interventions to low-income patients requiring only texting capabilities to send the blood glucose values, and a simple phone call to receive titration instructions. This intervention requires only a low-cost basic mobile phone, not a smart phone or internet access, which our patients often do not have."

## METHODS

**3a) CONSORT: Description of trial design (such as parallel, factorial) including allocation ratio**

Yes

"Objectives
The objectives of this study are to (1) evaluate if MITI is clinically effective by helping patients reach their optimal dose of insulin glargine (defined in outcome measures section), (2) determine if the intervention is feasible within the setting and population, (3) assess patient satisfaction with the intervention, and (4) measure the costs associated with this intervention."

**3b) CONSORT: Important changes to methods after trial commencement (such as eligibility criteria), with reasons**

Yes

"Prior to May 1st, 2014, patients were also stratified by HbA1c level (8-11% or > 11%). We removed this stratification after finding that not all patients had an HbA1c value available in their medical record at the time of enrollment."

"Since this is a pilot study, we have made adjustments to our technology to ensure the intervention runs smoothly. We discovered early in the trial that the initial health management platform used could not send text messages to prepaid mobile phones. Those patients affected by this issue either continued to attend in-person clinic appointments for insulin titration or were provided a mobile phone to use for the duration of the study. We resolved this issue in May 2014 when we switched to a different health management platform that could send text messages to any mobile phone."

**3b-i) Bug fixes, Downtimes, Content Changes**

**4a) CONSORT: Eligibility criteria for participants**

Yes

"It is implicit that a patient must be able to operate a mobile phone to participate in the study."

**4a-i) Computer / Internet literacy**

**4a-ii) Open vs. closed, web-based vs. face-to-face assessments:**

Yes

"The RA screens the patient for eligibility, explains the study, and provides them with a consent form to read and sign in person in the Adult Primary Care Center."

**4a-iii) Information giving during recruitment**

**4b) CONSORT: Settings and locations where the data were collected**

Yes
“Our research assistant (RA) collects data (such as fasting blood glucose readings, insulin dosage adjustments, and appointment duration) from patients after appointments, in-person or by phone, as well as appointment duration from clinicians.”
“The RA also arranges to administer the Diabetes Treatment Satisfaction Questionnaire, either when the patient is present in the clinic for their blood test or via phone.”

**4b-i) Report if outcomes were (self-)assessed through online questionnaires**

No, outcomes were not assessed through online questionnaires.

**4b-ii) Report how institutional affiliations are displayed**

**5) CONSORT: Describe the interventions for each group with sufficient details to allow replication, including how and when they were actually administered**

**5-i) Mention names, credential, affiliations of the developers, sponsors, and owners**

**5-ii) Describe the history/development process**

**5-iii) Revisions and updating**

**5-iv) Quality assurance methods**

**5-v) Ensure replicability by publishing the source code, and/or providing screenshots/screen-capture video, and/or providing flowcharts of the algorithms used**

**5-vi) Digital preservation**

**5-vii) Access**

Yes
"Patients in the MITI arm sign up for a web-based health management platform in the clinic with the assistance of the research staff."

**5-viii) Mode of delivery, features/functionalities/components of the intervention and comparator, and the theoretical framework**

Yes
“The platform allows patients and clinicians to communicate via text messaging. Patients receive a text message each weekday from the platform saying “What was your blood glucose reading this morning?” The messages are automatically delivered each morning at a time pre-specified by the patient, in either English or Spanish. Patients respond via text message with their fasting blood glucose level.”
“The diabetes nurse educator checks patients’ responses on the secure web portal each weekday…”

**5-ix) Describe use parameters**

**5-x) Clarify the level of human involvement**

**5-xi) Report any prompts/reminders used**

Yes
“Patients receive a text message each weekday from the platform saying “What was your blood glucose reading this morning?” The messages are automatically delivered each morning at a time pre-specified by the patient, in either English or Spanish.”

**5-xii) Describe any co-interventions (incl. training/support)**

Our study does not include any co-interventions.

**6a) CONSORT: Completely defined pre-specified primary and secondary outcome measures, including how and when they were assessed**

Yes
“Outcome measures
The primary outcome is whether or not a patient reaches his/her optimal insulin glargine dose within 12 weeks of enrolling in the study. Optimal insulin glargine dose is defined as the dose at which the patient has at least one fasting blood glucose value within the range of 80 to 130 mg/dL inclusive or the maximum dose that can be safely administered to the patient. The research staff records whether a patient has reached their optimal insulin dose at the time of the patient’s weekly titration phone call (if in the MITI arm) or clinic appointment (if in the usual care arm).
We hypothesize that the MITI arm will have a greater proportion of patients who reach their optimal insulin dose than the usual care arm. Other clinical effectiveness outcomes include the time taken to reach optimal dose, the incidence of hypoglycemia, and the change in HbA1c levels at baseline and three months after study enrollment.
Feasibility measures include patients’ text message response rate, ability of the diabetes nurse educator to reach patients for insulin titration, and the time spent by the diabetes nurse educator on the intervention.
We are measuring patient treatment satisfaction at baseline and three months after study enrollment using the Diabetes Treatment Satisfaction Questionnaire. We are using an additional questionnaire (the “change” version of the Diabetes Treatment Satisfaction Questionnaire) to measure the change in the patient’s treatment satisfaction between baseline and three months [15]. We are also using a semi-structured interview to gather qualitative feedback from patients in the MITI arm. This is administered when the patient has completed the intervention (by reaching their optimal insulin dose or when 12 weeks elapse).
We are collecting data on the costs of insulin titration to compare the intervention to the established standard of care in the clinic. These outcomes include the time spent by patients traveling to the clinic, time spent in the waiting room prior to the appointment, number and duration of insulin titration appointments, patient co-pays, and patient healthcare utilization (the number of non-insulin-related medical clinic visits at Bellevue made during the 12-week study period).”

**6a-i) Online questionnaires: describe if they were validated for online use and apply CHERRIES items to describe how the questionnaires were designed/deployed**

**6a-ii) Describe whether and how “use” (including intensity of use/dosage) was defined/measured/monitored**

**6a-iii) Describe whether, how, and when qualitative feedback from participants was obtained**

**6b) CONSORT: Any changes to trial outcomes after the trial commenced, with reasons**

No changes were made.

**7a) CONSORT: How sample size was determined**

**7a-i) Describe whether and how expected attrition was taken into account when calculating the sample size**

**7b) CONSORT: When applicable, explanation of any interim analyses and stopping guidelines**
Yes
“Data monitoring
A data safety monitoring board is designated to meet quarterly (or sooner if necessary) to discuss any potential safety issues. In particular, the study team and the board review any cases of hypoglycemia or hyperglycemia that occurs among our patients (to assess the safety of our titration algorithm) and our ability to reach patients by phone (to ensure that our patients are being monitored regularly during the intervention). “

**8a) CONSORT: Method used to generate the random allocation sequence**

Yes
“The random allocation sequence was computer –generated by a co-investigator and concealed in pre-sealed envelopes.”

**8b) CONSORT: Type of randomisation; details of any restriction (such as blocking and block size)**
Yes
“Patients are block randomized (block size of 4) to either arm of the study in a 1:1 ratio and stratified by whether the patient is initiating insulin therapy or having their existing insulin dose titrated. Prior to May 1st, 2014, patients were also stratified by HbA1c level (8-11% or > 11%). We removed this stratification after finding that not all patients had an HbA1c value available in their medical record at the time of enrollment.”

**9) CONSORT: Mechanism used to implement the random allocation sequence (such as sequentially numbered containers), describing any steps taken to conceal the sequence until interventions were assigned**
Yes
“The random allocation sequence was computer –generated by a co-investigator and concealed in pre-sealed envelopes. The patient’s arm assignment is revealed to the patient and research team at the time of study enrollment by the RA.”

**10) CONSORT: Who generated the random allocation sequence, who enrolled participants, and who assigned participants to interventions**
Yes
“The random allocation sequence was computer –generated by a co-investigator and concealed in pre-sealed envelopes. The patient’s arm assignment is revealed to the patient and research team at the time of study enrollment by the RA.”

**11a) CONSORT: Blinding - If done, who was blinded after assignment to interventions (for example, participants, care providers, those assessing outcomes) and how**

**11a-i) Specify who was blinded, and who wasn’t**
Yes
“The research team and clinicians are not blinded since the intervention requires that they alter the patient’s treatment plan or, in the case of the usual care arm, collect data from clinicians and patients regarding their appointments.”

**11a-ii) Discuss e.g., whether participants knew which intervention was the “intervention of interest” and which one was the “comparator”**

**11b) CONSORT: If relevant, description of the similarity of interventions**
This subitem is not relevant to our trial.

**12a) CONSORT: Statistical methods used to compare groups for primary and secondary outcomes**
Yes
“Data analysis
First, we will summarize all the baseline and follow-up measures using means (SD), medians (IQR), or frequencies, and then compare them between the two arms.  Second, we will test if baseline characteristics (including demographic measures and baseline HbA1c levels) are balanced between the two randomized arms.  Third, to evaluate the intervention effects, we will use the Chi-square test or Fisher’s exact test for categorical outcomes (including the primary outcome, whether or not a patient reaches optimal dose), Student’s t test or Wilcoxon rank sum test for continuous outcomes (such as change in HbA1c levels, rate of hypoglycemia, scores on the Diabetes Treatment Satisfaction Questionnaire, number and duration of titration appointments, and patient healthcare utilization), and log-rank test for time to reach optimal dose. Fourth, we will conduct multiple linear regression analyses for continuous outcomes and multiple logistics regression analyses for categorical outcomes to further evaluate the intervention effects, adjusting for some baseline characteristics and/or their interactions with the treatment assignment. At this stage, we will conduct multiple-imputation to deal with the missing data problem.  Finally, we will conduct descriptive analysis for other secondary outcomes such as feasibility outcomes, patient travel time and waiting room time, and patient co-pays. We will also review the content of semi-structured interviews to identify common themes in patient feedback.”

**12a-i) Imputation techniques to deal with attrition / missing values**

Yes
“At this stage, we will conduct multiple-imputation to deal with the missing data problem.”

**12b) CONSORT: Methods for additional analyses, such as subgroup analyses and adjusted analyses**
Yes
“Fourth, we will conduct multiple linear regression analyses for continuous outcomes and multiple logistics regression analyses for categorical outcomes to further evaluate the intervention effects, adjusting for some baseline characteristics and/or their interactions with the treatment assignment.”

RESULTS

**13a) CONSORT:  For each group, the numbers of participants who were randomly assigned, received intended treatment, and were analysed for the primary outcome**
This item is not relevant because this article is a study protocol. Results are not yet available.

**13b) CONSORT:  For each group, losses and exclusions after randomisation, together with reasons**
This item is not relevant because this article is a study protocol. Results are not yet available.

**13b-i) Attrition diagram**

**14a) CONSORT: Dates defining the periods of recruitment and follow-up**
Yes
"Recruitment for this study occurred between June 2013 and December 2014. We continue to collect intervention and follow-up data from our patients who are currently enrolled. The results of our data analysis are expected to be available in April 2015."

**14a-i) Indicate if critical “secular events” fell into the study period**

**14b) CONSORT: Why the trial ended or was stopped (early)**
This item is not relevant because our trial was not stopped early and has not ended.

**15) CONSORT: A table showing baseline demographic and clinical characteristics for each group**
This item is not relevant because this is an ongoing study protocol. Results are not yet available.

**15-i) Report demographics associated with digital divide issues**
This item is not relevant because this is an ongoing study protocol. Results are not yet available.

**16a) CONSORT: For each group, number of participants (denominator) included in each analysis and whether the analysis was by original assigned groups**

**16-i) Report multiple “denominators” and provide definitions**

This item is not relevant because this is an ongoing study protocol. Results are not yet available.

**16-ii) Primary analysis should be intent-to-treat**

**17a) CONSORT: For each primary and secondary outcome, results for each group, and the estimated effect size and its precision (such as 95% confidence interval)**

This item is not relevant because this is an ongoing study protocol. Results are not yet available.

**17a-i) Presentation of process outcomes such as metrics of use and intensity of use**

**17b) CONSORT: For binary outcomes, presentation of both absolute and relative effect sizes is recommended**

This item is not relevant because this is an ongoing study protocol. Results are not yet available.

**18) CONSORT: Results of any other analyses performed, including subgroup analyses and adjusted analyses, distinguishing pre-specified from exploratory**

This item is not relevant because this is an ongoing study protocol. Results are not yet available.

**18-i) Subgroup analysis of comparing only users**

**19) CONSORT: All important harms or unintended effects in each group**

We have not encountered harm in this study. The results of the qualitative feedback are not yet available.

**19-i) Include privacy breaches, technical problems**

**19-ii) Include qualitative feedback from participants or observations from staff/researchers**

## DISCUSSION

**20) CONSORT: Trial limitations, addressing sources of potential bias, imprecision, multiplicity of analyses**

**20-i) Typical limitations in ehealth trials**

Yes

“The MITI study has a few limitations. We anticipate a small sample size for this study, which will limit our statistical power. We also have to consider volunteer bias, since those patients who choose to participate may not be representative of the population of patients we are seeking to treat (those with type II diabetes in need of insulin titration).

While we use a strong study design (randomized controlled trial), our study is not blinded. Since our intervention directly affects the treatment our patients receive in the clinic, the patient’s primary care provider and their other clinicians may become aware that their patient is participating in the study. In addition, those who are allocated to the usual care arm will be contacted periodically by the research staff for data collection. While this data collection is necessary to measure certain outcomes for both study arms, we cannot rule out that patients may alter their behavior since they are aware that their treatment progress is being monitored (observer bias).”

**21) CONSORT: Generalisability (external validity, applicability) of the trial findings**

**21-i) Generalizability to other populations**

**21-ii) Discuss if there were elements in the RCT that would be different in a routine application setting**

**22) CONSORT: Interpretation consistent with results, balancing benefits and harms, and considering other relevant evidence**

**22-i) Restate study questions and summarize the answers suggested by the data, starting with primary outcomes and process outcomes (use)**

This item is not relevant because this is an ongoing study protocol. Results are not yet available.

**22-ii) Highlight unanswered new questions, suggest future research**

## Other information

**23) CONSORT: Registration number and name of trial registry**

Yes

Trial Registration

clinicialtrials.gov ID # NCT01879579

**24) CONSORT: Where the full trial protocol can be accessed, if available**

Not available.

**25) CONSORT: Sources of funding and other support (such as supply of drugs), role of funders**

Yes

“We would like to thank our funders, the New York University – Health and Hospitals Corporation Clinical and Translational Science Institute (NYU-HHC CTSI) for the 2013 CTSI grant #UL1 TR000038 and the 2014 H-3 Research Grant award.”

**X26-i) Comment on ethics committee approval**

**x26-ii) Outline informed consent procedures**

**X26-iii) Safety and security procedures**

**X27-i) State the relation of the study team towards the system being evaluated**
