# Supplementary material for: Addendum to: The Mobile Insulin Titration Intervention (MITI) for Insulin Glargine Titration in an Urban, Low-Income Population: Randomized Controlled Trial Protocol
Source: JMIR Res Protoc. 2015 Dec 21;4(4):e138. doi: 10.2196/resprot.5403 (PMC6318147; doi:10.2196/resprot.5403)
Supplement: Supplementary file 1 [file resprot_v4i4e138_app2.pdf]

Insulin glargine titration algorithm

Guidelines for weekly titration

- Titration typically occurs on Thursdays.
- Titration is restricted by the lowest fasting blood glucose value.
- Patients with a lowest fasting blood glucose value ≤ 200 need at least 3 values from the current week (Monday to Thursday) to change the dose.
- Patients with a lowest fasting blood glucose value > 200 need at least 2 values from the current week (Monday to Thursday) to change the dose.

Fasting blood glucose values (mg/dL)	Increase/decrease in insulin dose (IU)
> 220	+ 5
181 - 220	+ 4
151 - 180	+ 3
131 - 150	+ 2
80 - 130	0
75 – 79	- 1
< 75	- 2

< 65	- 4
Severe hypoglycemia requiring assistance from others or < 60	- 6
